# Effect of presenting robot hand stiffness to human arm on human-robot collaborative assembly tasks

**DOI:** 10.3389/frobt.2025.1660691

**Published:** 2025-10-30

**Authors:** Junya Yamamoto, Kenji Tahara, Takahiro Wada

**Affiliations:** 1 Human Robotics Laboratory, Nara Institute of Science and Technology, Ikoma, Japan; 2 Department of Mechanical Engineering, Kyushu University, Fukuoka, Japan

**Keywords:** human-robot collaboration, human-robot interactions, human-machine teaming, human-machine interface, assembly task, robotics

## Abstract

In response to the growing need for flexibility in handling complex tasks, research on human–robot collaboration (HRC) has garnered considerable attention. Recent studies on HRC have achieved smooth handover tasks between humans and robots by adaptively responding to human states. Collaboration was further improved by conveying the state of the robot to humans via robotic interactive motion cues. However, in scenarios such as collaborative assembly tasks that require precise positioning, methods relying on motion or forces caused by interactions through the shared object compromise both task accuracy and smoothness, and are therefore not directly applicable. To address this, the present study proposes a method to convey the stiffness of the robot to a human arm during collaborative human-robot assembly tasks in a manner that does not affect the shared object or task, aiming to enhance efficiency and reduce human workload. Sixteen participants performed a collaborative assembly task with a robot, which involved unscrewing, repositioning, and reattaching a part while the robot held and adjusted the position of the part. The experiment examined the effectiveness of the proposed method, in which the robot’s stiffness was communicated to a participant’s forearm. The independent variable, tested within-subjects, was the stiffness presentation method, with three levels: without the proposed method (no presentation) and with the proposed method (real-time and predictive presentations). The results demonstrated that the proposed method enhanced task efficiency by shortening task completion time, which was associated with lower subjective workload scores.

## 1 Introduction

The transition from mass production to diverse, small-scale production has posed challenges such as increased production costs and demand for flexibility in industrial processes (Okimoto and Niitsuma, 2020). Although industrial robots excel in repetitive and precise tasks, their limited adaptability to dynamic production lines renders them unsuitable for tasks involving intricate and variable designs (Okimoto and Niitsuma, 2020). In contrast, collaborative robots designed to work alongside humans without safety fences have gained attention because of their ability to combine human adaptability with robotic efficiency (Kildal et al., 2018; Joseph et al., 2020; Okimoto and Niitsuma, 2020). This growing interest in human–robot collaboration (HRC) is evident from the appearance of safety requirements in the ISO standards, specifically for industrial robot systems (ISO 10218-1:2011, 2011; ISO 10218-2:2011, 2011).

**FIGURE 1:**
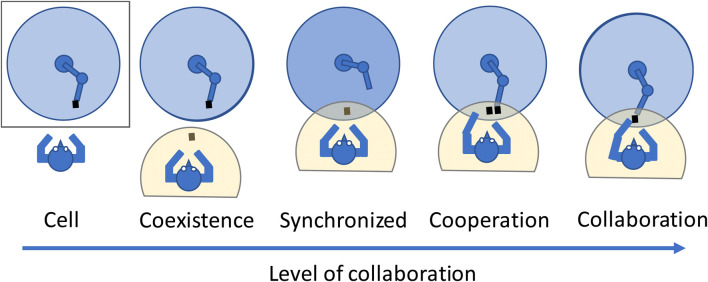
Various levels of cooperation between a human worker and a robot. Redrawn based on the conceptual framework described in (Wilhelm et al., 2016; International Federation of Robotics IFR, 2020).

Conventional industrial robots are usually operated without direct human contact, separated by safety fences. In contrast, collaborative robots are designed to work effectively in close proximity to or in direct physical contact with humans (Kildal et al., 2018). A previous study (Wilhelm et al., 2016) defined the types of interactions between a human and a robot as illustrated in Figure 1. These include “coexistence,” wherein there are no fences but the workspace is not shared. They also include “cooperation,” wherein the workspace is shared but the occupants (human and robot) do not simultaneously handle the same products or components (Wilhelm et al., 2016; Kildal et al., 2018). At these levels safety functions such as safety-rated monitored stops or protective stops triggered by safeguarding devices (ISO 10218-1:2011, 2011) are typically employed to prevent physical contact between a human and a robot. In contrast, “collaboration” involves simultaneous handling of the same products or components by humans and robots, which has recently gained significant attention in the field of HRC. The present research focuses on this “collaboration,” aiming to deepen the understanding of how humans and robots can efficiently and fluidly perform tasks while manipulating the same object together.

Safety (ISO 10218-1:2011, 2011; ISO 10218-2:2011, 2011; Kildal et al., 2018; Joseph et al., 2020; Schepp et al., 2022) is a fundamental prerequisite for HRC. Once safety is ensured, many studies focus on enhancing efficiency by enabling robots to recognize human states (Kupcsik et al., 2018; Pan et al., 2019; Mohammed and Wada, 2023; Mohammed et al., 2024). However, collaborative work with robots can potentially impose cognitive and physical burdens on humans. This is particularly the case for humans who lack a full understanding of the force, speed, movement direction, and actions of the robot (Segura et al., 2021), or who do not trust the robot as a competent team member (Mukherjee et al., 2022). These challenges are particularly evident in industrial settings such as factories, where humans are required to interact with diverse and unfamiliar types of robots. Therefore, to achieve safe and efficient collaboration, ensuring human physical safety, building trust, and alleviating human workload by intuitively conveying the robot’s intentions are imperative.

Several studies have been actively conducted on object handover as examples of HRC. Moon et al. (2014) proposed a method to convey the robot’s handover target position to humans through the robot’s gaze, enabling humans to acquire the object faster before the robot arrives at the handover position. Okimoto and Niitsuma (2020) suggested utilizing sound to indicate the robot’s destination to humans, which would enable humans to move faster before the robot arrives at its destination. Maccio et al. (2022) concluded that visualizing the forthcoming actions of a robot using mixed reality devices could facilitate the interaction and result in fewer collisions. Previous studies (Mohammed and Wada, 2023; Mohammed et al., 2024) demonstrated that presenting the robot’s future handover position to humans via a vibrotactile armband improves task efficiency. These methods help humans perceive the robot’s future destination (Moon et al., 2014; Okimoto and Niitsuma, 2020) and actions of the robot (Maccio et al., 2022). Such perception enhances work efficiency and reduces human workload during the motion planning phase preceding physical contact with the robot.

In collaboration, however, consideration of the phase involving physical contact becomes crucial, rather than relying solely on recognition of the robot’s state before the contact. For example, in handover (Costanzo et al., 2021) or assembly (Bonilla and Asada, 2014) tasks, issues such as danger or discomfort may arise if one party forcibly pulls the object while the other holds it or if one party releases the object prematurely. Such issues may result from a lack of recognition of the mechanical state of the robot. Therefore, in collaboration, the mechanical state is deemed a crucial element because it is challenging to perceive visually and can significantly impact performance. Costanzo et al. (2021) proposed a method in which a robot slightly retracts its hand just before pulling it back when holding an object together with a human. This indicates that the robot securely holds the object and is ready to take over it. This method communicates the robot’s intention through the force resulting from the dynamic interaction between the human and the robot. Additionally, an existing study (Yamamoto et al., 2024) proposed a method that conveys the internal mechanical states of a robot, such as mechanical impedance, which emerge before any interaction force arises. This approach demonstrated smoother handover, which can be further enhanced by incorporating predicted future state changes.

The present study focuses on collaborative assembly tasks. In certain situations, conveying the mechanical state of a robot via an object, as in Costanzo et al. (2021), may not be feasible. For example, when the robot’s interaction force or movement inadvertently alters the shared object’s position or orientation, this can be undesirable for precise positioning. In our preliminary study, Yamamoto et al. (2024) proposed a method that conveys the robot’s internal states to humans through sensory augmentation without affecting the shared object or task. Building on this approach, the present study explores its potential applicability to collaborative assembly scenarios. While the method in Yamamoto et al. (2024) has demonstrated its effectiveness in handover tasks, its application to collaborative assembly tasks remains underexplored.

Therefore, the purpose of the present study is to develop a method to convey the robot’s mechanical impedance to humans via tightening forces during collaborative human-robot assembly tasks where both a human and a robot engage with the same products or components simultaneously. The present study aims to investigate the effectiveness of the method to enhance efficiency and reduce human workload through human-in-the-loop experiments.

The remainder of this paper is organized as follows. Section 2 introduces a haptic presentation method for humans to intuitively discern the robot’s mechanical impedance. Section 3 presents an experiment to evaluate the effectiveness of the proposed method in an assembly task. Finally, Section 4 discusses the findings of this study and future research direction.

## 2 Methods

### 2.1 Conveying stiffness of robots to humans

Smooth collaboration between humans and robots requires effective communication between the two distinct agents to comprehend each other’s intentions or states. We convey the robot’s intentions through non-verbal information, as verbal communication may introduce further delays. Given the importance of the robot’s mechanical state in collaboration, where humans and robots jointly grasp a single component, this state is deemed crucial, because it is challenging to visually perceive and it significantly affects the performance.

Leveraging the robot’s stiffness—an internal state measurable before interaction—is advantageous for promptly responding to changes in the robot’s mechanical state. In contrast, relying on gripping force, which is detectable only after interaction, delays the response. Consequently, understanding the robot’s intention is equated with understanding the robot’s stiffness in this paper.

**FIGURE 2:**
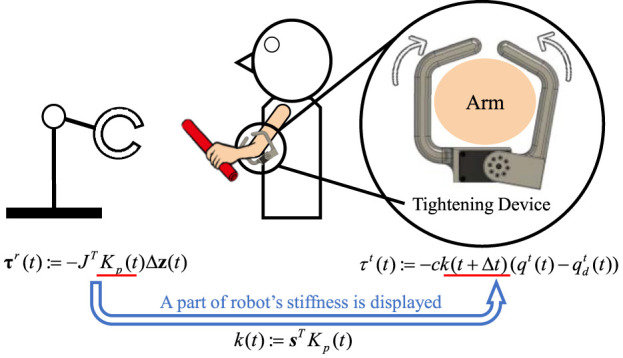
Schematic diagram illustrating how the robot’s stiffness $K_{p}\left(t\right)$ is presented to a human. A specific direction of the stiffness, denoted by $k\left(t\right)$ , is mapped to the tightening device. In this study, $s:={\left[0,1,0\right]}^{T}$ was used.

The stiffness of the robot’s end-effector was conveyed to the human through the stiffness changes corresponding to the tightening or loosening of a device attached to the human forearm (Figure 2). The rationale for selecting the compressive force to convey the state is because tactile sense requires the shortest reaction period among visual, auditory, and tactile senses (Chan and Ng, 2012). Additionally, this method is particularly useful even when humans are visually occupied, which is often the case in HRC scenarios (Mohammed et al., 2024).

The tightening device, weighing 160 g, comprises a DC motor (XM430-W210-T, Dynamixel) and gripping components fabricated using a 3D printer. We experimented with various materials, including rubber bands; however, the elasticity of rubber significantly impeded presentation speed compared to rigid acrylonitrile butadiene styrene (ABS). The torque of the tightening device was determined as follows:

$$\tau^{t}\left(t\right):=-c\cdot k\left(t+\Delta t\right)\;\left(q^{t}\left(t\right)-q_{d}^{t}\left(t\right)\right),$$ (1)

where the scalar *k*(*t*) denotes the stiffness of the robot end-effector in a certain direction within the task coordinates, which will be described later, and $q^{t}\left(t\right)$ and $q_{d}^{t}\left(t\right)$ represent the measured and desired angles of the tightening device, respectively. The scalar *c* acts as a scaling factor that maps the stiffness of the robot end-effector *k*(*t*) to the equivalent stiffness of the tightening device $c\cdot k\left(t\right)$ . The scalars *c* and $q_{d}^{t}\left(t\right)$ were determined according to the experimental task as described in the following subsection. Additionally, $\Delta t\;\left(\geq 0\right)$ was introduced to advance the timing of communicating the robot’s state by a few seconds to compensate for the human reaction time, as research (Fujita et al., 2010) has demonstrated that presenting signals immediately before the robot’s action can reduce human cognitive load.

The present study posits the following hypotheses:

H1 Presenting the robot’s stiffness through compression on the forearm will facilitate the recognition of the robot’s mechanical state, which is difficult to visually perceive, thereby reducing the workload and improving work efficiency.

H2 Advancing the timing of presenting the robot’s stiffness by Δt will reduce the delay in responding to variations in the mechanical state of the robot, thereby improving work efficiency.

To validate these hypotheses, we conducted experiments in assembly tasks. Additionally, objective evaluations such as reaction time and subjective evaluations such as workload were performed.

### 2.2 Robot

**FIGURE 3:**
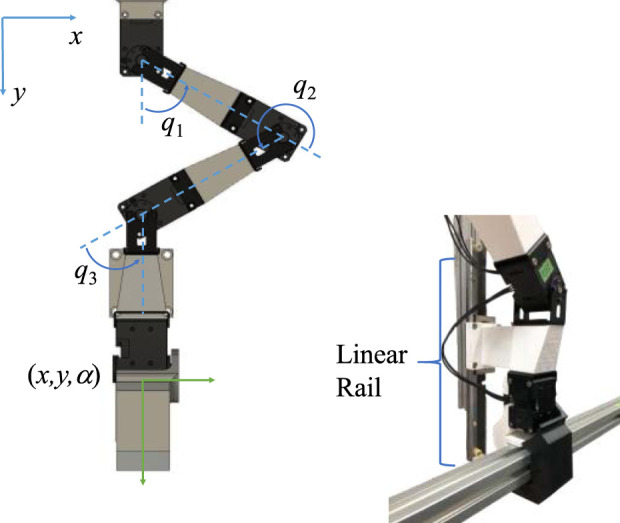
Planar 3-DoF robot arm.

As depicted in Figure 3, a 3-degree-of-freedom (DOF) planar robot was utilized for the experiment, and the motion of the end-effector was mechanically restricted to one DOF in the vertical direction using a linear rail. Task-space position control in Equation 2 was employed for the robot:

$$\begin{array}{l} \tau :=-J^{T}\left(q\right)K_{p}\left(z\left(t\right)-z_{d}\right)-K_{v}\dot{q}\left(t\right)\\ z\left(t\right):={\left[x\left(t\right),y\left(t\right),\alpha \left(t\right)\right]}^{T},z_{d}:={\left[0,y_{d},0\right]}^{T}, \end{array}$$ (2)

where $q\left(t\right)={\left[q_{1},q_{2},q_{3}\right]}^{T}\left(\in R^{3}\right)$ denotes the joint angles of the robot. In this context, ***z*** (*t*) represents the position and orientation of the robot hand in task coordinates and $\alpha \;\left(=q_{1}+q_{2}+q_{3}\right)$ indicates the orientation. Here, α=0 when the longitudinal direction of the hand is aligned with the *y*-axis, as in Figure 3. Additionally, $K_{p}:=\text{diag}\left[k_{x},k_{y},k_{\alpha }\right]$ represents the stiffness matrix of the end-effector in the task coordinates. In the present study, the stiffness in the *y*-direction was conveyed to a human, meaning that $k={\left[0,1,0\right]}^{T}K_{p}=k_{y}$ was employed in Equation 1 as the motion of the end-effector was restricted along the *y* direction. Furthermore, $k_{x}$ and $k_{\alpha }$ were fixed to zero, as they had no physical significance under the one-DOF constraint of the end-effector motion. Note that the desired value of y , denoted as $y_{d}$ , varied over time according to the task.

### 2.3 Task scenario

**FIGURE 4:**
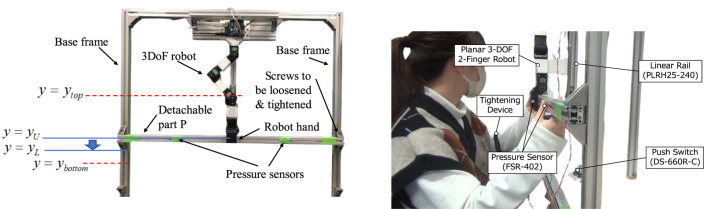
Experimental setup (left) and a participant in the assembly task (right). The top image shows the location of the detachable part (P) at the end of the preparation phase in the downward condition. From this state, the participant loosens the screws, moves the part P to $y=y_{L}$ with both hands, releases it with robotic assistance, and then tightens the screws again. Finally, the robot returns to $y_{top}$ . Pressure sensors measure the timing of the participant’s grasping and releasing.

This experiment, illustrated in Figure 4, centers on an assembly task involving collaborative positional adjustments of a metal frame facilitated by a robot manipulator. The task involves changing the installation height of the detachable part (designated as P in the figure), which is affixed horizontally to the base frames with screws. Movement of part P is required from a higher position $y=y_{U}$ to a lower position $y=y_{L}$ (downward condition), or conversely, from $y=y_{L}$ to $y=y_{U}$ (upward condition). It was assumed that the robot lacked prior knowledge of the target location for relocating the detachable part.

Given that the detachable part was secured to the base frames with screws, it was imperative to first unscrew them, relocate the part, and then reattach it. The robot held the object so that the human could remove their hands from the detachable part. This allowed the use of both hands to loosen and tighten the screws, thus improving work efficiency.

**FIGURE 5:**
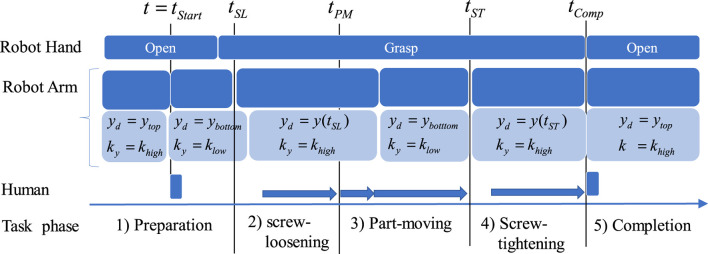
Phases in the task and how robot and human collaborate.

The task details are as follows (also refer to Figures 4, 5). For clarity, this description predominantly addresses the downward condition, as the upward condition is nearly identical.

1. Preparation phase: Initially, the robot arm was at a starting position ( $y=y_{top}$ ). When a human pressed a button, the robot moved to the location of the detachable part, depicted by $y=y_{U}$ , by setting the desired position for the robot’s position control to $y_{d}=y_{bottom}$ and the stiffness of the arm to low ( $k_{y}=k_{low}$ ). After a few seconds pause, the robot hand closed and grasped the detachable part.
2. Screw-loosening phase: This phase started at time $t_{SL}$ , which occurred 2–3 s after the robot grasped the object. During this phase, the robot adopted a high end-effector stiffness ( $k_{y}=k_{high}$ ) and set its desired position to $y_{d}=y\left(t_{SL}\right)$ , which corresponded to the robot’s position at the moment it started to hold the object. In this phase, the human could remove their hands from the detachable part P and loosen the screws with both hands. Here, it is noted that $y\left(t_{SL}\right)$ ideally matches $y_{U}$ . However, the observed value $y\left(t_{SL}\right)$ is used because the position of the detachable part inevitably involves uncertainty, due to dimensional tolerances of the assembled components. In addition, the position of the end effector when grasping the detachable part is not uniquely determined, as it depends on where the part is grasped. To ensure that the end effector remains nearly at the same position even when the screw is released under such uncertainty, we set the reference to the observed position after grasping, $y\left(t_{SL}\right)$ .
3. Part-Moving phase: The human grasped the detachable part with both hands and lowered it to $y=y_{L}$ , then halted. Initially, the robot endeavored to maintain its current position with high stiffness. When the human applied a 4 mm downward movement (or 1 mm upward movement for the upward condition), the robot lowered its stiffness ( $k_{y}=k_{low}$ ). Then, the desired position was updated to $y_{d}=y_{bottom}$ , thereby facilitating movement of the object and robot by the human. As previously mentioned, it was presumed that the robot was unaware of the desired location of the part ( $y_{L}$ ); thus, it had to be determined by the operator’s actions. In the experiment, tapes indicating $y=y_{L}$ were affixed to the base frames. Participants were instructed to align the detachable part with this tape prior to commencing the experiment.
4. Screw-Tightening phase: This phase initiated at time $t=t_{ST}$ , defined as the moment when the robot’s hand stopped moving. This was determined when the changes in the position obtained from the joint sensors remained within a certain range over a given period. During this phase, the robot set its desired position as $y_{d}:=y\left(t_{ST}\right)$ and adopted high end-effector stiffness ( $k_{y}=k_{high}$ ). Subsequently, the human released their hands from the part and tightened the screws to secure the part to the base frame.
5. Completion phase: When the human pressed the button again, the robot opened the hand, released the detachable part, and returned to the initial position ( $y_{d}=y_{top}$ ).

The present study employed the following setting $y_{top}$ = 0.196 m, $y_{bottom}$ = 0.302 m, $y_{U}$ = 0.24 m, and $y_{L}$ = 0.28 m (Figure 4).

The values for the stiffness $k_{y}$ used in the object-holding and compliant modes are set as follows.

$k_{low}$ : The stiffness level at which the robot alone cannot secure the object in a fixed position, enabling easy movement when released by the human. In this study, $k_{low}$ = 0.1 was used.

$k_{high}$ : The stiffness level at which the robot alone can sustain the object in a fixed position even if the human releases their hands from it. In the present study, $k_{high}$ = 70 was used.

### 2.4 Experimental design

This experiment employed a within-subjects design where each participant experienced all three levels of the robot hand stiffness presentation method, which was introduced as the independent variable. The three conditions tested were as follows:

- No Presentation (No): Participants did not wear the tightening device, and the task was executed without any knowledge of the robot’s stiffness change.
- Current Presentation (Current): Participants wore the tightening device, and the real-time stiffness of the robot arm ( Δt = 0 s) was communicated.
- Future Presentation (Future): Participants wore the tightening device, and the future stiffness of the robot arm after a given time ( Δt = 0.25 s) was presented, aimed at compensating for the delay in human reaction time. The value of 0.25 s was determined through a brief pilot test for the present assembly task, based on a previous study (Tanaka et al., 2012), which demonstrated that the typical human reaction time to haptic stimuli ranges from 0.2 to 0.25 s. The order of presenting the three levels of experimental conditions was counterbalanced.

### 2.5 Participants

Sixteen participants aged 21–30 years (13 males and 3 females) provided informed consent and were involved in the experiment approved by the Ethics Review Committee for Human Research at Nara Institute of Science and Technology (2023-I-33). The participants were compensated with approximately $10 for their participation.

### 2.6 Experimental design

**FIGURE 6:**
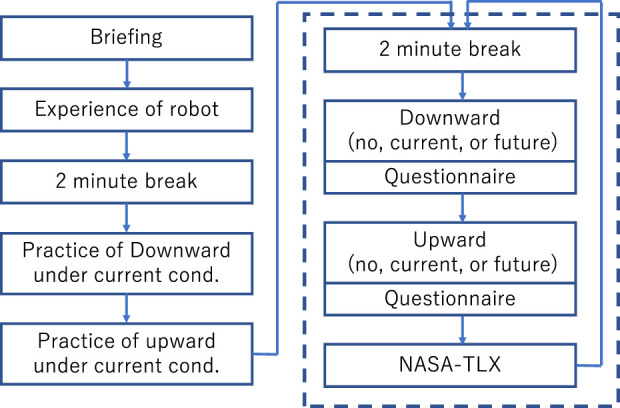
Experimental procedure for human–robot assembly. The procedures depicted inside the dotted square were repeated three times, one for each condition: No, Current, and Future.

The procedure of this experiment is illustrated in Figure 6. Initially, the participants were briefed on the experiment, including the procedures and data collection, and provided informed consent. The participants were informed that the tightening device conveyed the stiffness of the robot arm, while the timing of the stiffness presentation was not mentioned. After a 2-min break, the participants wore the tightening device and practiced the task while experiencing changes in the stiffness of the robot arm under the Current condition. This practice continued until participants felt confident in performing the task adequately. Following another 2-min break, the participants completed detachable object movement tasks with the assistance of the robot for one of the three stiffness-presentation method conditions, as described in Section 2.2, twice: first with downward movement of the detachable object and then with upward movement. After each task execution, the participants completed a questionnaire to assess the various subjective aspects of the task as mentioned in the following sections. Additionally, the NASA-TLX questionnaire was completed after both trials of each stiffness-presentation method condition were finished. Similarly, for the remaining two stiffness-presentation method conditions, participants performed two trials—one downward movement and one upward movement of the detachable object—followed by completing the questionnaire after each trial. The NASA-TLX questionnaire was completed after both trials for each stiffness-presentation method condition were finished. As described earlier, the order of these conditions was counterbalanced and varied for each participant.

Participants were instructed not to grasp the detachable part during the screw-loosening and screw-tightening phases. In the part-moving phase, they were instructed to hold the section of the part where the pressure sensor was attached while moving the part. They were also instructed to complete the tasks as quickly as possible.

### 2.7 Evaluation method

#### 2.7.1 Objective evaluation by temporal indices

**FIGURE 7:**
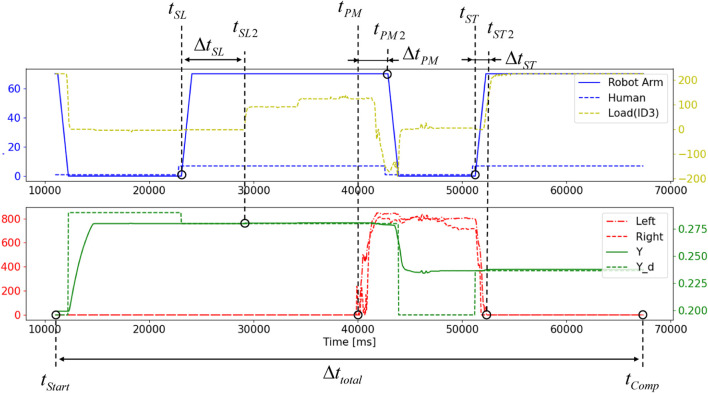
Four temporal indices to evaluate the effectiveness of the proposed method to the collaborative task is illustrated with signals related to states of robot, human, as well as their interactions. Please refer the text of the paper for the detail.

To investigate the smoothness of task execution, the following four temporal indices are introduced (Figure 7).

1. Screw-loosening timing ( $\Delta t_{SL}$ ): This is defined as $\Delta t_{SL}:=t_{SL2}-t_{SL}$ , where $t_{SL}$ denotes the time the robot enters position-holding mode in the screw-loosening phase, as already defined in the previous section. The time $t_{SL2}$ is defined as the time when the human begins to loosen the screw, indicated by the slight downward movement of the detachable object due to gravitational force.
2. Part-moving timing ( $\Delta t_{PM}$ ): This is defined as $\Delta t_{PM}:=t_{PM2}-t_{PM}$ , where $t_{PM}$ is defined as the time when both hands start to grasp the part, measured by the pressure sensors. The time $t_{PM2}$ is defined as the time when the robot becomes compliant ( $k_{y}=k_{low}$ ) to be moved by human hands.
3. Screw-tightening timing ( $\Delta t_{ST}$ ): This is defined as $\Delta t_{ST}:=t_{ST2}-t_{ST}$ . Here, $t_{ST}$ is defined as the time when the robot enters position-holding mode in the screw-tightening phase, as already defined earlier, and $t_{ST2}$ is defined as the time when both human hands release the part for starting to tighten one of the screws, measured by the pressure sensors attached to the detachable part P.
4. Total Task Completion Time ( $\Delta t_{total}$ ): This is defined as $\Delta t_{total}:=t_{Comp}-t_{Start}$ , where $t_{Start}$ is defined as the time when the human first pushes the button in the preparation phase to start the task, and $t_{Comp}$ is defined as the time when the human pushes the button in the completion phase to signal the completion of the task.

A trial of the task is judged as ‘failure’ when the detachable part P is dropped at least once during the trial. There are two opportunities to ‘fail’ in a trial: just before the screw-loosening and screw-tightening phases, during which both hands are released from the detached part. Releasing the part before entering each of these phases, i.e., before the robot’s stiffness switches to $k_{y}=k_{high}$ , it is judged as ‘failure.’ If a trial is judged as ‘failure,’ the data regarding the temporal indices of the participants are replaced by the mean value over successful participants.

#### 2.7.2 Subjective evaluations

We evaluated participants’ perception of trust in the robot, clarity of the presentation of robot stiffness, subjective smoothness of the task, and anxiety about dropping the detachable part. For this purpose, the questionnaire items Q1–Q5 listed in Table 1 were administered using a Visual Analog Scale (VAS). The left and right sides of the VAS were labeled “strongly disagree” and “strongly agree,” respectively. Scores of 0 and 100 were assigned to the left and right ends for Q1 through Q4, and inversely for Q5.

**TABLE 1:** Questionnaire items (Q1–Q5) used in the subjective evaluations.

Id	Questionnaire item
Q1	Were you able to trust the robot to perform the task?
Q2	Was your experience working with the robot one of dependable performance?
Q3	Was it clear when the stiffness of the robot’s arm changed?
Q4	Was it clear when the stiffness of the robot’s arm changed?
Q5	Did you feel anxious about dropping the metal part?

For Q5, “Strongly Disagree” was assigned a score of 100, and “Strongly Agree” was assigned a score of 0. For all other questions, “Strongly Disagree” was assigned a score of 0, and “Strongly Agree” was assigned a score of 100. Additionally, the workload for each presentation method was assessed using the Japanese version (Haga and Mizukami, 1996) of the NASA-TLX (Hart and Staveland, 1988).

## 3 Results

Under the No presentation condition, wherein the proposed method was not utilized, a total of six failures were observed—three during screw-loosening in the downward direction and three during screw-tightening in the upward direction. Conversely, no failures were noted under the Current presentation condition, where the current stiffness of the robot end-effector was provided to participants. In the Future presentation condition, one failure occurred during the screw-tightening phase in the upward direction.

For data in which normality and homoscedasticity were not rejected, a one-way repeated measures analysis of variance (ANOVA) was used to investigate the main effects, followed by *post hoc* paired t-tests with Bonferroni correction. Otherwise, Friedman tests followed by *post hoc* Wilcoxon signed-rank tests with Bonferroni correction were applied. The results for each evaluation index are presented in the following subsections, while the details of the *post hoc* statistical analyses are summarized in Table 2.

**TABLE 2:** Overview of statistical results.

Temporal indices		Future vs. current				Future vs. no				Current vs. no				Test
Condition	Indices		*p*	*df/n*	*r/d*		*p*	*df/n*	*r/d*		*p*	*df/n*	*r/d*	
Down	Screw-loosening	-	-	-	-	-	-	-	-	-	-	-	-	n/a
	Part-moving	-	-	-	-	-	-	-	-	-	-	-	-	n/a
	Screw-tightening	*	0.043	16	−0.54	***	0.000	16	−0.88	***	0.000	16	−0.88	WSR
	Task completion	-	1.0	16	0.12	†	0.050	16	−0.53	**	0.008	16	−0.67	WSR
Up	Screw-loosening	-	-	-	-	-	-	-	-	-	-	-	-	n/a
	Part-moving	-	-	-	-	-	-	-	-	-	-	-	-	n/a
	Screw-tightening	**	0.009	16	−0.66	***	0.000	16	−0.85	*	0.011	16	−0.65	WSR
	Task completion	-	1.0	15	−0.063	**	0.009	15	−0.79	*	0.012	15	−0.76	Paired t
Questionnaire														
Condition	Question No.													
Down	Q1	-	0.49	13	0.28	**	0.003	16	0.72	*	0.043	16	0.54	WSR
	Q2	*	0.026	13	0.67	**	0.008	15	0.73	*	0.049	15	0.56	
	Q3	-	0.13	10	0.56	***	0.000	16	0.88	***	0.000	16	0.79	
	Q4	-	0.11	11	0.55	**	0.002	15	0.84	*	0.027	16	0.59	
	Q5	-	0.41	14	−0.30	-	0.076	16	0.49	*	0.023	16	0.60	
Up	Q1	-	1.0	14	0.12	***	0.000	16	0.88	***	0.000	16	0.87	
	Q2	-	1.0	13	0.048	***	0.000	16	0.87	***	0.000	16	0.87	
	Q3	-	0.62	12	0.25	***	0.000	16	0.88	***	0.000	16	0.88	
	Q4	-	0.11	14	0.12	***	0.000	16	0.88	***	0.000	16	0.87	
	Q5	-	0.12	14	−0.48	***	0.000	16	0.84	***	0.000	16	0.88	
WWL		-	1.0	15	−0.09	**	0.006	15	−0.85	*	0.011	15	−0.78	Paired t

Statistical significances indicate the proposed method outperforms the “No” condition, and the Future condition outperforms the Current condition. The “test” column shows the tests (“WSR” for Wilcoxon signed-rank test and “paired t” for paired t-test). “*df*/*n*” shows degrees of freedom (*df*) for the paired t-test and the sample size (*n*) for the Wilcoxon test, while “*d*/*r*” column indicates effect size (Cohen’s *d* or *r*). See the main text for details. † p < 0.1, *p < 0.05, **p < 0.01, ***p < 0.001.

### 3.1 Temporal indices

**FIGURE 8:**
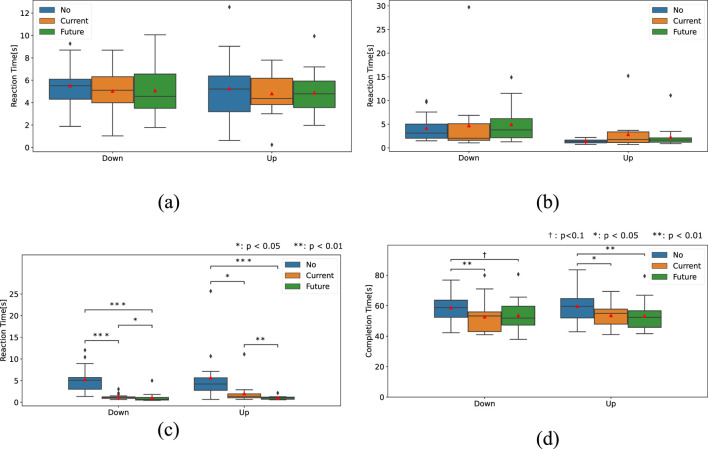
Results of four temporal indices. **(a)** Screw-loosening timing ( $\Delta t_{SL}$ ). **(b)** Part-moving timing ( $\Delta t_{PM}$ ). **(c)** Screw-tightening timing ( $\Delta t_{ST}$ ). **(d)** Total task completion time.

Figure 8a through (d) illustrate the four temporal indices for each presentation method condition.

#### 3.1.1 Screw-loosening time

A one-way repeated measures ANOVA was used to analyze the screw-loosening time. The results revealed no significant difference in both downward (*p* = 0.63) and upward (*p* = 0.73) scenarios.

#### 3.1.2 Part-moving time

Friedman tests for part-moving time also revealed no significant difference in both downward (*p* = 0.087) and upward scenarios (*p* = 0.066).

#### 3.1.3 Screw-tightening time

Regarding screw-tightening time, the Friedman test revealed significant differences in both downward (*p* < 0.001) and upward (*p* < 0.001) scenarios.

As shown in Table 2, the *post hoc* test showed that screw-tightening time was significantly longer under the No condition than under both conditions using the stiffness presentation device in both downward and upward scenarios. It also revealed that the time under the Future condition was significantly shorter than that under the Current condition in both scenarios.

#### 3.1.4 Task completion time

For the total task completion time, the Friedman test and ANOVA revealed significant differences in the downward (*p* =0.0030) and upward (*p* =0.0033) conditions, respectively.

Post-hoc tests showed that, in the downward scenario, the No condition resulted in a significantly longer time than the Current condition and a marginally longer time than the Future condition. In the upward scenario, the No condition was significantly longer than both conditions using the stiffness presentation device.

### 3.2 Subjective indices

**FIGURE 9:**
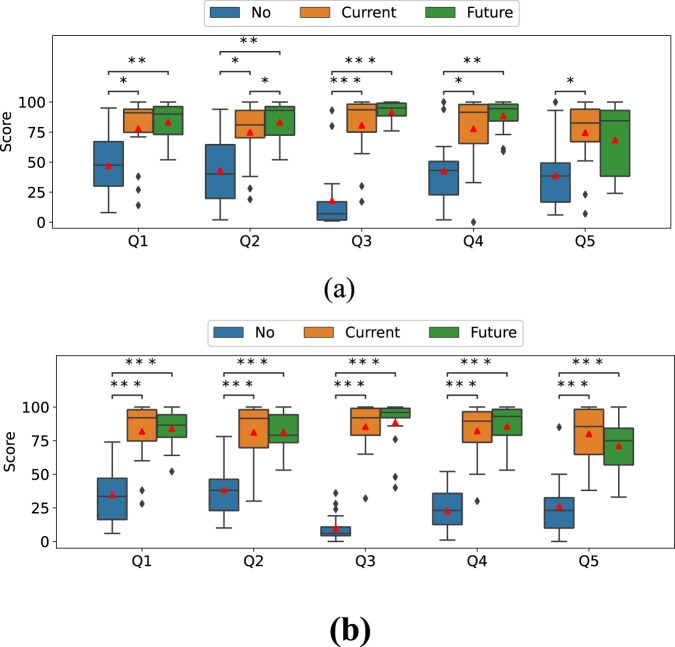
Questionnaire on Q1 on trust in the robot, Q2 on feeling secure with the robot, Q3 on ease of understanding the timing of the change in stiffness of the robot arm, Q4 on smoothness of the work, and Q5 on anxiety about dropping the metal frame. **(a)** Downward scenario. **(b)** Upward scenario.

Figures 9a,d depict the results of the five questionnaires in the downward and upward scenarios, respectively. Friedman tests for Q1 through Q5 revealed the statistical significance of the main effect of the stiffness-presentation method factor (Table 3).

**TABLE 3:** Results of Friedman test for questionnaires.

Condition	Q1	Q2	Q3	Q4	Q5
Downward	*	**	***	**	*
Upward	***	***	***	***	***

*p < 0.05, **p < 0.01, ***p < 0.001.

As shown in Table 2, *post hoc* tests for Q1, Q3, and Q4 revealed that the No condition had significantly lower scores than both the Future and Current conditions, while no significant difference was observed between the Future and Current conditions in both the downward and upward scenarios.

For Q2, the No condition was significantly lower than both the Future and Current conditions in both downward and upward scenarios. However, unlike the other questions, the Future condition was also significantly higher than the Current condition in the upward scenario, while no significant difference was found between them in the downward scenario.

For Q5 (downward), the Current condition scored higher than the No condition, while no significant difference was found between the No and Future conditions or between the Current and Future conditions. The upward scenario followed the same pattern as the other questions.

**FIGURE 10:**
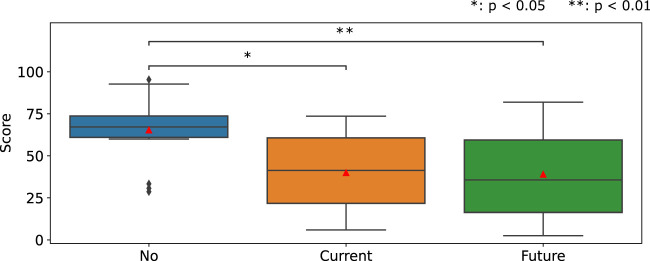
WWL score of NASA-TLX for human-robot assembly.

Figure 10 illustrates the WWL score of the NASA-TLX. The one-way repeated measure ANOVA revealed a significant main effect of the stiffness-presenting method factor (*p* = 0.00047) on the WWL score. The paired-t tests with Bonferroni correction showed that the WWL score under the No condition was significantly higher than those in both conditions using the stiffness presentation device.

## 4 Discussion and conclusion

The significant decrease in the total task completion time and the screw-tightening time in the Current and Future conditions demonstrates the effectiveness of the proposed method, which presents the robot’s stiffness to the human forearm via the tightening device. A significant difference was observed in the screw-tightening time under the Current condition; however, no significant difference was found in the screw-loosening time under the same condition. This can be interpreted as follows: the onset of the screw-loosening time is determined solely by the robot’s behavior, making it relatively straightforward for human operators to predict, whereas the onset of the screw-tightening time depends on the interaction between the human and robot, which introduces additional complexity and makes it more challenging for humans to understand. The proposed method was found to be effective in such situations, where determining whether a transition to the next phase had occurred is challenging. This successful enhancement of human–robot collaboration efficiency is evident regardless of the presentation method (Current or Future). While the reduction in total task time around a few seconds may not be large, it is not negligible in the context of repetitive industrial operations. In particular, the reduction in screw-tightening time represents a substantial relative improvement, indicating that the proposed method can effectively enhance performance in key subprocesses. Furthermore, the significant decrease in subjective workload is noteworthy. The ability of the proposed method to reduce workload while maintaining—or even slightly improving—temporal performance is especially important for industrial applications, since high workload conditions often cause fatigue and reduced task performance or quality. Furthermore, the results of the subjective evaluations indicated a significant increase in all scores of the Q1–Q5 questionnaires for the Current and Future conditions.

This finding suggests that presenting the stiffness of the robot to the human clearly conveyed the invisible robot stiffness (Q3), thereby may have enhanced trust (Q1) and reliance (Q2) in robot coworkers. This is also reflected in the decrease in anxiety regarding failure (Q5), which lead to the subjective smoothness of collaboration (Q3) and potentially contributes to the shorter task completion times. The enhanced smoothness of collaboration is evident from the trend in the WWL score of the NASA-TLX. In conclusion, the results suggest that the timing of object transfer became clearer by using a tightening device to communicate the stiffness of the robot arm, facilitating the perception of changes in the challenging mechanical states of the robot arm. It is also suggested that this improvement leads to enhanced task efficiency through reductions in task completion time and screw-tightening time, as well as reduced workload, thereby providing evidence in support of hypothesis H1.

As observed from the results, providing cues in advance (Future condition) resulted in a further significant reduction in screw-tightening time for both the downward and upward movement conditions compared to cues provided in the Current condition. However, no significant difference was observed in the total task completion time, suggesting that the reduction in time by communicating the future information was insufficient to affect the total task time. Therefore, H2, which concerns additional enhancement of task efficiency with the Future over the Current condition, was not supported in terms of overall task efficiency, while improvements were observed in a sub-process (i.e., screw-tightening time).

Okimoto and Niitsuma (2020) conducted a study on HRC by focusing on the motion planning phase until physical contact with a robot was established; however, they excluded the phase of physical interaction. Their findings revealed that presenting the current state of the robot using an auditory signal reduces human workload, while providing information regarding future movements enables humans to initiate actions early. Thus, one of the contributions of the present study is that it is the first to show that this knowledge can be extended to the phase of physical interaction with a robot by using tactile signals. This extension was accomplished by presenting the stiffness of the robot arm, which was associated with improved collaborative task efficiency, reduced subjective workload scores, and enhanced subjective trust ratings in the robot. Furthermore, presenting the stiffness of the robot in advance could reduce the delay in response to changes in its mechanical state.

In the preliminary research for the present study, we investigated the effectiveness of presenting the mechanical state of a robot hand during a task focused on a simple handover–takeover of objects between a human and a robot (Yamamoto et al., 2024). The results demonstrated that although subjective trust rating was enhanced, the improvement in task effectiveness was limited, as evaluated by temporal indices. In contrast, the present study builds upon these findings to examine the applicability of this approach to a collaborative assembly task. Notably, applying the method proposed in object-handover research (Yamamoto et al., 2024) to assembly tasks is not straightforward. This is because the type of information that needs to be conveyed varies depending on given tasks, requiring careful design considerations regarding which internal states should be communicated. In fact, in Yamamoto et al. (2024), the robot conveyed changes in its object grasping state via stiffness information of the robot ‘hand’ to enhance the smoothness of the handover process. In contrast, given the requirements of the assembly task, the present study redesigned the method to convey the robot’s ability to hold an object or to be moved by an external human force using the robot arm’s stiffness in a specific direction. As a result, the present study demonstrated not only the feasibility of applying this approach to assembly tasks but also a significant improvement in task efficiency, specifically through reductions in task completion time and screw-tightening time. The findings suggest that presenting robot stiffness to humans is particularly beneficial in complex, multistep tasks where precise coordination is essential. Furthermore, research (Mart et al., 2025) has demonstrated the effectiveness of a new device that combines the communication of the stiffness of the robot (Yamamoto et al., 2024) with a robot-intended handover position conveyed by vibrotactile stimuli (Mohammed et al., 2024), specifically for handover tasks. Although this approach may not be directly applicable to the assembly tasks targeted in the present study, combining the proposed method with other approaches holds promise for further improving the efficiency of assembly tasks. This can be a potential direction for future research.

The present research has a few limitations. First, the validation of the proposed method was limited to a single type of assembly task, involving only two conditions of stiffness changes. Consequently, it remains uncertain whether the proposed method is applicable to tasks involving multiple or continuous levels of stiffness change. However, a preliminary study (Yamamoto et al., 2024) showed that the same method can be applied to a simple handover–takeover task in which the stiffness of the robot changes across four levels. Therefore, the benefits of this method can potentially be extended to similar tasks, particularly those in which changes in impedance are crucial for collaboration. Further research is required in this field. Furthermore, given individual differences in human perception and behavior, caution should be exercised when generalizing the present findings, and further validation with a larger and more diverse sample is recommended. It should also be noted that several findings, such as improvements in trust and perceived clarity, relied on subjective ratings. While subjective measures are inherently vulnerable to bias, in the present study they were consistent with temporal indices, implying that the subjective ratings provided valid reflections of participants’ experiences. Nevertheless, future work should incorporate a broader range of behavioral indicators to strengthen the link between subjective and behavioral evidence, because in the present study only temporal indices were employed as behavioral data.

Moreover, we did not investigate individual characteristics such as arm diameter dimensions or just noticeable differences for force sensation on the arm. For instance, concerning the Δt timing of the Future presentation condition, an optimal value is considered to exist for each participant. Consequently, determining Δt for each individual is crucial in elucidating the performance of the future presentation method. Furthermore, as the experiments lasted only for an hour, the effectiveness of this presentation method during prolonged use and the time required for users to become accustomed to it have not been verified.

Additionally, the present study utilized force or haptic sensation to convey the stiffness information of the robot owing to its rapid transmission (Chan and Ng, 2012). However, the effectiveness of this communication method using other sensory modalities, including visual or auditory signals, remains unclear. It should be noted that the proposed method using haptic signals is particularly useful even when humans are visually occupied, which is often the case in HRC scenarios (Mohammed et al., 2024). Finally, the primary objective of this study was to verify the effects of conveying the state of a robot to humans, thereby limiting its ability to comprehend human behavior. By integrating efforts to enhance robot intelligence (for example, Costanzo et al. (2021)) with the proposed method, more advanced collaboration can be achieved, representing a crucial direction for future research.

To conclude, the contributions of the present study are 2-fold: (1) to introduce a method for conveying the stiffness of a robot to a human in a manner that does not affect the shared object or task, particularly in collaborative assembly tasks requiring accurate positioning, and (2) to demonstrate through human-in-the-loop experiments that the proposed method could reduce the workload and improve task efficiency by decreasing the time required for task completion. Furthermore, the proposed method may be applicable to a broader range of applications in tasks requiring close human–robot collaboration, such as precise manufacturing processes or tasks involving dynamic adjustments to shared objects, where both humans and robots interact with a single object simultaneously. By addressing the challenges of conveying mechanical states without affecting task execution, the proposed method could serve as a foundation for future research to explore its applicability to diverse collaborative scenarios.

## Data Availability

The raw data supporting the conclusions of this article will be made available by the authors, without undue reservation.
